# Aberrant Dynamic Functional Connectivity of Posterior Cingulate Cortex Subregions in Major Depressive Disorder With Suicidal Ideation

**DOI:** 10.3389/fnins.2022.937145

**Published:** 2022-07-19

**Authors:** Weicheng Li, Chengyu Wang, Xiaofeng Lan, Ling Fu, Fan Zhang, Yanxiang Ye, Haiyan Liu, Kai Wu, Guohui Lao, Jun Chen, Guixiang Li, Yanling Zhou, Yuping Ning

**Affiliations:** ^1^The Affiliated Brain Hospital of Guangzhou Medical University, Guangzhou, China; ^2^The First School of Clinical Medicine, Southern Medical University, Guangzhou, China; ^3^Guangdong Engineering Technology Research Center for Translational Medicine of Mental Disorders, Guangzhou, China; ^4^School of Biomedical Sciences and Engineering, South China University of Technology, Guangzhou, China; ^5^Guangdong Institute of Medical Instruments, Guangzhou, China; ^6^Institute of Biological and Medical Engineering, Guangdong Academy of Sciences, Guangzhou, China

**Keywords:** major depressive disorder, posterior cingulate cortex, magnetic resonance imaging, suicidal ideation, dynamic functional connectivity (dFC)

## Abstract

Accumulating evidence indicates the presence of structural and functional abnormalities of the posterior cingulate cortex (PCC) in patients with major depressive disorder (MDD) with suicidal ideation (SI). Nevertheless, the subregional-level dynamic functional connectivity (dFC) of the PCC has not been investigated in MDD with SI. We therefore sought to investigate the presence of aberrant dFC variability in PCC subregions in MDD patients with SI. We analyzed resting-state functional magnetic resonance imaging (fMRI) data from 31 unmedicated MDD patients with SI (SI group), 56 unmedicated MDD patients without SI (NSI group), and 48 matched healthy control (HC) subjects. The sliding-window method was applied to characterize the whole-brain dFC of each PCC subregion [the ventral PCC (vPCC) and dorsal PCC (dPCC)]. In addition, we evaluated associations between clinical variables and the aberrant dFC variability of those brain regions showing significant between-group differences. Compared with HCS, the SI and the NSI groups exhibited higher dFC variability between the left dPCC and left fusiform gyrus and between the right vPCC and left inferior frontal gyrus (IFG). The SI group showed higher dFC variability between the left vPCC and left IFG than the NSI group. Furthermore, the dFC variability between the left vPCC and left IFG was positively correlated with Scale for Suicidal Ideation (SSI) score in patients with MDD (i.e., the SI and NSI groups). Our results indicate that aberrant dFC variability between the vPCC and IFG might provide a neural-network explanation for SI and may provide a potential target for future therapeutic interventions in MDD patients with SI.

## Introduction

Suicide presents a heavy burden on public health, resulting in nearly one million deaths each year worldwide (Turecki and Brent, 2016). Important contributors to suicide include familial, social, cultural, genetic vulnerability, psychological, and psychiatric factors (Turecki and Brent, 2016). It is reported that up to 80% of people who die by suicide have mental disorders (Ilgen et al., 2010) and more than half of suicide attempters suffered from depression at the time of the attempt (Chahine et al., 2020). Suicidal ideation (SI), described as the consideration or plan to commit suicide (Klonsky and May, 2014), is a significant risk factor for suicide among patients with major depressive disorder (MDD) (Klonsky et al., 2016). Therefore, efforts to achieve a better comprehension of the neurobiological mechanisms underlying SI in patients with MDD are crucial to make progress in the treatment and prevention of suicide.

The posterior cingulate cortex (PCC), which forms a key part of the default mode network (Buckner et al., 2008), demonstrates different brain activity and increased functional connectivity during the resting state than during cognitive tasks (Greicius et al., 2003; Pfefferbaum et al., 2011). The PCC forms a key hub for self-referential processing (Johnson et al., 2009), cognitive control (Vanyukov et al., 2015), and emotion processing and underlies multidomain cognitive functions by linking to distal cortical areas, such as the prefrontal cortex (Leech et al., 2011). In the last decade, much neuroimaging literature has reported structural and functional changes in the PCC of MDD patients with SI (Schmaal et al., 2020). A structural study found increased PCC volume in MDD patients with SI when compared with MDD patients with suicide attempts (SAs) (Hong et al., 2021). Functional magnetic resonance imaging (fMRI) has been widely used to investigate aberrant brain activity in the PCC in MDD patients with SI, and brain dysfunction has been related to cognitive control (Minzenberg et al., 2015) and self-referential (Quevedo et al., 2016) observations in these patients. For instance, Marchand et al. (2013) reported aberrant functional connectivity between the PCC and dorsolateral prefrontal cortex and inferior frontal gyrus (IFG) during motor control tasks in MDD patients with SI. Additionally, this aberrant functional connectivity was positively correlated with SI intensity (Marchand et al., 2013). Analogous to this, decreased resting-state functional connectivity between the PCC and habenula has also been detected in MDD patients with SI (Ambrosi et al., 2019).

The abovementioned studies were conducted from the viewpoint that the PCC is a single homogeneous structure; however, accumulating evidence indicates that the PCC is not homogeneous, either structurally or functionally (Leech et al., 2011). On the basis of the cytoarchitectonic characteristics of the PCC, Fan et al. (2016) recommended that the PCC should be divided into two major subregions, the ventral PCC (vPCC) and dorsal PCC (dPCC) nuclei. The dPCC is reported to play an important role in the orientation of the self and body in visual space (Vogt et al., 2006), whereas the vPCC is at an intermediate stage of information processing between visual recognition and emotion-related substrate and plays a key role in self-reflective function (Johnson et al., 2002; Uddin et al., 2005). PCC subregion-based network abnormalities or volume differences have been reported in schizophrenia (Ebisch et al., 2018), epilepsies (Parvizi et al., 2021), autism spectrum disorders (Lau et al., 2019), obsessive-compulsive disorder (Matsumoto et al., 2010), Alzheimer’s disease (Xu et al., 2009), and chronic pain (Yoshino et al., 2018). Nevertheless, PCC dysfunction at the subregional level has been little studied in MDD patients with SI. Therefore, we still know little about whether PCC subregion-based dysfunction is disrupted in MDD patients with SI.

Using the approach of static functional connectivity, aberrant brain activity in PCC subregions was reported in MDD patients with SI (Chase et al., 2021, 2017). Of note, resting-state functional connectivity has traditionally relied on static analytic approaches that assume stable patterns of connectivity across the entire resting scan period. However, human brain connectivity shows time-varying profiles across periods of unconstrained rest (Allen et al., 2014; Zalesky et al., 2014). Analysis of the variability of functional connectivity (dFC) may therefore enable a more sophisticated demonstration of the spontaneous fluctuating nature of neural signals (Vidaurre et al., 2021) and their association with cognition and behavioral performance (Kucyi et al., 2017). Thus, investigation from the perspective of temporal dynamics is needed to explore aberrant dFC in MDD patients with SI. Recently, dFC is increasingly being suggested as a prognostic indicator of disease (Preti et al., 2017; Lurie et al., 2020), such as Parkinson’s disease (Kim J. et al., 2017), Huntington’s disease (Espinoza et al., 2019), and depression (Liao et al., 2018). Moreover, a prior study reported that patients with depression with SI revealed increased dynamic connectomics relative to patients with depression without SI and healthy controls (HCs) (Liao et al., 2018). Thus, a better understanding of dFC variability may offer nuanced insights into brain activity in MDD patients with SI, further improving our understanding of the psychopathological mechanisms underlying MDD with SI. Up to now, no study has investigated dFC variability differences in PCC subregions in MDD patients with SI.

In the current study, we analyzed resting-state fMRI data from 31 unmedicated MDD patients with SI, 56 unmedicated MDD patients without SI, and 48 matched healthy subjects. The sliding-window method was applied to characterize the whole-brain dFC of each PCC subregion. We generated the following hypotheses: (i) relative to HCs and MDD patients without SI, MDD patients with SI would exhibit anomalous dFC patterns in PCC subregions; and (ii) the aberrant dFC variability would show associations with clinical variables. With these hypotheses, we sought to identify aberrant dFC variability in PCC subregions in MDD patients with SI. In addition, we evaluated correlations between clinical variables and the aberrant dFC variability of brain regions showing significant between-group differences.

## Materials and Methods

### Participants

In total, 89 unmedicated patients with MDD between the ages of 18 and 65 years were drawn from the Molecular Biomarkers of Antidepressant Response study (clinical trial number: ChiCTR1800017626) cohort, the data of which were published in our previous study (Lan et al., 2021). For all patients, the entrance criteria were (i) meeting the criteria for MDD according to the Diagnostic and Statistical Manual of Mental Disorders, 5th edition; (ii) available imaging data and data on symptoms; (iii) a score ≥ 17 on the 17-item Hamilton Depression Rating Scale (HAMD-17) (Helmreich et al., 2012); and (iv) medication-free for at least 4 weeks before inclusion in the trial.

The exclusion criteria included a history of other major psychiatric disorders meeting the criteria of axis I of the Diagnostic and Statistical Manual of Mental Disorders, 5th edition, current serious and unstable somatic disease or a history of neurologic or other chronic medical conditions, a history of substance abuse or dependence, breast-feeding, and pregnancy. Recruitment was carried out at the Affiliated Brain Hospital of Guangzhou Medical University, Guangzhou, China. Ethics approval was obtained from the ethics committees of the Affiliated Brain Hospital of Guangzhou Medical University. In addition, healthy volunteers (*n* = 48) recruited from the local community served as HCs. Informed consent was signed by all participants before participating in this study.

### Assessment of Suicidal Ideation and Depression

The severity of depressive symptoms was assessed using the 17-item HAMD. All raters were masters- or doctoral-level psychiatrists who had undergone training on performing the HAMD-17 before the study to maintain inter-rater reliability, and they all showed an intra-class correlation coefficient > 0.9. The Scale for Suicidal Ideation (SSI) was used to assess the presence and intensity of SI according to 19 items (Beck et al., 1979). Each item has three alternative statements graded from 0 to 2, with the total score ranging from 0 to 38 points, with higher scores indicating greater SI. In this study, the patients with MDD were classified into an SI group (SSI > 3) and a no SI (NSI) group (SSI ≤ 3). This threshold has been described as a clinically significant cutoff for SI in previous studies (Holi et al., 2005; Ballard et al., 2015; Grunebaum et al., 2018).

### Magnetic Resonance Imaging Data Acquisition

Participants underwent resting-state fMRI on a 3T Philips Achieva MRI Scanner (Philips, Netherlands). Whole-brain fMRI was acquired using a gradient-echo echo planar imaging sequence with the following parameters: repetition time (TR) = 2,000 ms, echo time = 30 ms, flip angle = 90°, slice thickness = 4 mm, number of slices = 33, and field of view = 220 mm × 220 mm. A total of 240 functional volumes were acquired in 8 min. During the MRI scans, all participants were instructed to keep their eyes closed but stay awake.

### Resting-State Functional Magnetic Resonance Imaging Preprocessing

Functional image preprocessing was performed using the Data Processing Assistant for Resting-State fMRI (DPARSF^1^) implemented in MATLAB (version R2013b). For each participant, the first 10 functional volumes were removed to ensure signal stabilization, then the remaining 230 volumes were corrected for timing differences between slices. The motion-corrected functional images were conducted using a six motion parameter (rigid body). Notably, the mean framewise displacement (FD) based on the Jenkinson model (FD-Jenkinson) was computed by averaging the FD from every time point for each participant (Jenkinson et al., 2002). Participants with more than 3 mm of head movement or 3° of rotation were excluded. The images were then spatially normalized to the standard Montreal Neurological Institute echo planar imaging template and resampled to 3 mm × 3 mm × 3 mm. After spatial normalization, the images were smoothed using a 4-mm full-width at half-maximum Gaussian kernel. Subsequently, we treated the six parameters from the rigid-body translation, the white matter signal, and the CSF signal as nuisance covariates to be regressed out. Finally, the images were filtered with a temporal band-pass filter of 0.01–0.08 Hz.

### Dynamic Functional Connectivity Analysis

**FIGURE 1:**
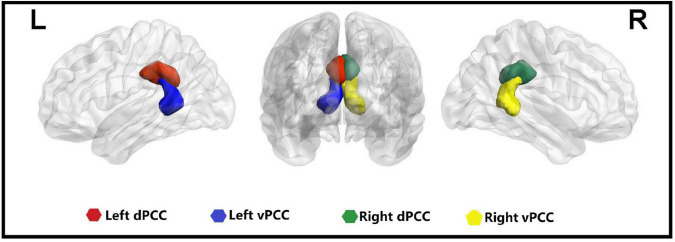
Four seeds of the posterior cingulate cortex in the bilateral hemisphere. L, left; R, right; dPCC, dorsal posterior cingulate cortex; vPCC, ventral posterior cingulate cortex.

Bilateral dPCC and bilateral vPCC regions of interest (ROIs) were derived from the Brainnetome Atlas (^2^ Figure 1). dFC analysis was conducted using a sliding-window approach in the DPABI software^3^. The sliding-window method was performed to explore time-varying changes in functional connectivity during resting-state fMRI scans. The resting-state blood oxygenation level-dependent (BOLD) time series was segmented into 50 TR windows with a size of 100 s. A sliding window with a step size of 1 TR was applied, resulting in 181 consecutive windows across the entire scan. We chose a window length of 50 TR (100 s) with a step size of 1 TR (2 s) because it has been shown to be able to maintain a balance between capturing rapidly shifting dynamic relationships and obtaining steady correlations (Leonardi and Van De Ville, 2015; Shunkai et al., 2021). For each window, correlation z maps were calculated between the truncated time course of the ROI and all other voxels using Fisher’s *z*−transformed Pearson correlation coefficient, resulting in 181 sliding-window correlation z maps across the entire scan for each participant. Consequently, the dFC was estimated by calculating the standard deviation (SD) of the z maps across the 181 windows, and *z*-standardization was then applied to the dFC maps. Finally, all dFC maps were spatially smoothed using a Gaussian kernel of 4 mm × 4 mm × 4 mm full-width at half maximum. To further validate the reliability of the results, we also analyzed other window sizes of 30 and 70 TR (Liao et al., 2014).

### Statistical Analyses

Demographic and clinical data were tested for normality using the Shapiro–Wilk or Kolmogorov–Smirnov normality test. If demographic and clinical data passed the normality test, a Student’s *t*-test or one-way ANOVA was used, whereas a Mann–Whitney test was performed if data were not normally distributed. Chi-square tests or Fisher exact tests were used for categorical variables. Statistical calculations were carried out using Statistical Package for the Social Sciences 24.0 (SPSS Inc., NY, United States).

To identify the within-group dFC patterns of each PCC subregion, one-sample *t*-tests were conducted in the SI, NSI, and HC groups (*p* < 0.05, uncorrected). For each PCC subregion, analysis of covariance (ANCOVA) was used to test for between−group differences in dFC maps within the union mask of one-sample *t*-tests of the SI, NSI, and HC groups. Age, gender, and mean FD were treated as covariates. All statistical maps were corrected for multiple comparisons using Gaussian random field (GRF) correction (cluster significance *p* < 0.05/4 = 0.0125, voxel significance *p* < 0.005) performed using DPABI software. The mean *z*-scores of brain regions showing significant differences among the three groups were extracted for further *post hoc* analyses (*p* < 0.05, Bonferroni correction test). Finally, correlations between clinical variables (SSI scores and HAMD without suicide) and the aberrant dFC variability measurements were performed in the patients with MDD using Spearman correlation (*p* < 0.05, Bonferroni-corrected test).

## Results

### Demographic and Clinical Characteristics

There were no significant differences in gender, mean FD, and age between the SI, NSI, and HC groups (all *p* > 0.05). In addition, no significant differences were found in education, duration of illness, and age of onset between the SI and NSI groups (all *p* > 0.05). However, we found significant differences in the scores of HAMD-17, HAMD-17 without suicide, and SSI between the SI and NSI groups (all *p* < 0.05). The detailed demographic and clinical features of the participants are presented in Table 1.

**TABLE 1:** Demographic and clinical features of subjects.

Variables	SI	NSI	HCs	*T/Z/F*/χ^2^	*P*-value
Numbers of subjects	33	56	48		–
Gender (male/female)	10/23	27/29	23/25	3.216	0.200^a^
Age (years)	24.52 ± 5.82	26.04 ± 5.13	27.54 ± 5.95	2.900	0.059^b^
Education (years)	13.09 ± 2.98	13.14 ± 3.00	NA	–0.079	0.937^c^
Duration of illness (month)	30.17 ± 24.06	23.26 ± 21.89	NA	1.618	0.106^d^
Age of onset	22.03 ± 6.26	24.21 ± 5.09	NA	–1.793	0.076^c^
HAMD-17	26.21 ± 5.32	22.21 ± 4.23	NA	3.685	0.001^c^**
HAMD-17 without suicide	23.61 ± 5.37	21.23 ± 4.21	NA	2.177	0.034^c^*
SSI	15.88 ± 5.69	0.95 ± 1.05	NA	7.971	<0.001^d^***
Mean framewise displacement	0.05 ± 0.02	0.05 ± 0.02	0.06 ± 0.02	0.419	0.658^b^

*SI, major depressive patients with suicidal ideation; NSI, major depressive patients without suicidal ideation; HCs, healthy controls; HAMD-17, the 17-item Hamilton Depression Rating Scale; SSI, scale for suicide ideation.*

*^a^Chi-square test.*

*^b^One-way ANOVA.*

*^c^Two-sample t-test.*

*^d^Mann–Whitney U test.*

*p < 0.05, **p < 0.01, ***p < 0.001.

### Dynamic Functional Connectivity Variability in the Posterior Cingulate Cortex Subregions

**FIGURE 2:**
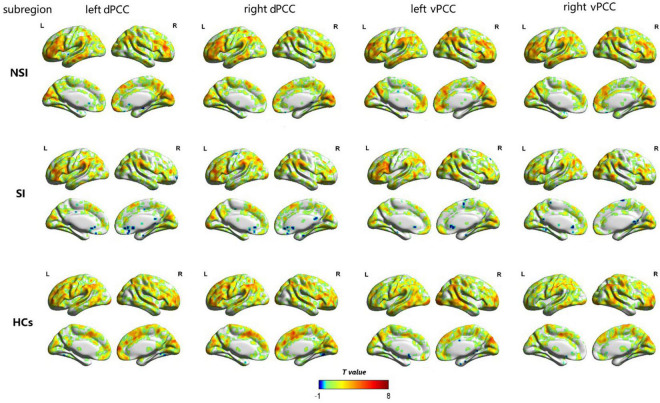
The dFC variability patterns of the bilateral dorsal posterior cingulate cortex (dPCC) and the bilateral ventral posterior cingulate cortex (vPCC) within NSI, SI and HCs groups (*p* < 0.05, uncorrected). The color bar represents a dynamic functional connection. dFC, dynamic functional connectivity; dPCC, dorsal posterior cingulate cortex; vPCC, ventral posterior cingulate cortex; SI, major depressive patients with suicidal ideation; NSI, major depressive patients without suicidal ideation; HCs, healthy controls.

**FIGURE 3:**
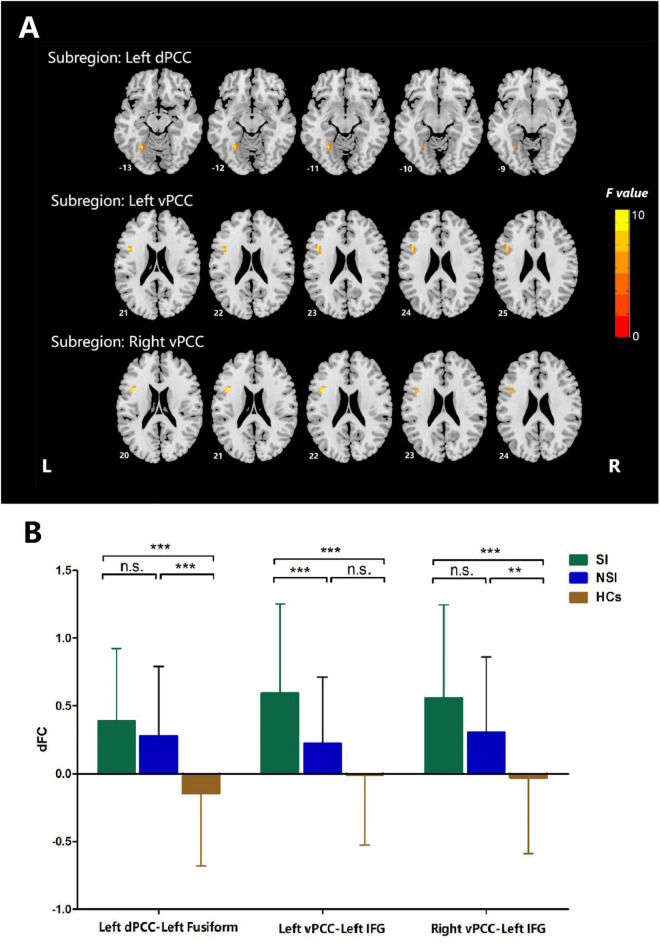
ANCOVA analyses of dFC values among three groups when taking the subregion of posterior cingulate cortex as a seed. **(A)** Brain regions with significant differences among three groups, voxel *p* < 0.005, cluster *p* < 0.0125, GRF corrected. **(B)** *Post hoc* analyses of dFC values among three groups. Bonferroni corrected. dFC, dynamic functional connectivity; vPCC, ventral posterior cingulate cortex; dPCC, dorsal posterior cingulate cortex; IFG, inferior frontal gyrus; SI, major depressive patients with suicidal ideation; NSI, major depressive patients without suicidal ideation; HCs, heathy controls. n.s., not significant. **p < 0.01, ***p < 0.001.

The dFC variability of each PCC subregion, as derived from the one-sample *t*-tests, is shown separately for the three groups in Figure 2 (*p* < 0.05, uncorrected). Significant differences in dFC variability between the three groups were observed between the left dPCC and left fusiform gyrus, left vPCC and left IFG, and right vPCC and left IFG (Table 2 and Figure 3A; GRF corrected, cluster significance *p* < 0.0125, voxel significance *p* < 0.005). However, no significant differences were found in the whole-brain dFC variability of the right dPCC between the three groups. The results of the *post hoc* analysis on the brain regions showing significant differences are shown in Figure 3B (*p* < 0.05, Bonferroni-corrected test). Compared with the HCs, the SI and NSI groups showed higher dFC variability between the left dPCC and left fusiform gyrus and between the right vPCC and left IFG. The SI group exhibited higher dFC variability between the left vPCC and left IFG than the NSI group.

**TABLE 2:** The areas of significantly different dFC among the SI, NSI, and HCs group (voxel *p* < 0.005, cluster *p* < 0.0125, GRF corrected).

Subregion	Significant regions	MNI coordinates			Voxel size (mm^3^)	*F*-value
		*X*	*Y*	*Z*		
Left dPCC	Left Fusiform	–27	–57	–12	486	15.345
Left vPCC	Left inferior frontal gyrus	–45	9	24	513	11.637
Right vPCC	Left inferior frontal gyrus	–42	9	21	459	11.122

*SI, major depressive patients with suicidal ideation; NSI, major depressive patients without suicidal ideation; HCs, healthy controls; dPCC, dorsal posterior cingulate cortex; vPCC, ventral posterior cingulate cortex.*

### Correlation Analyses

**FIGURE 4:**
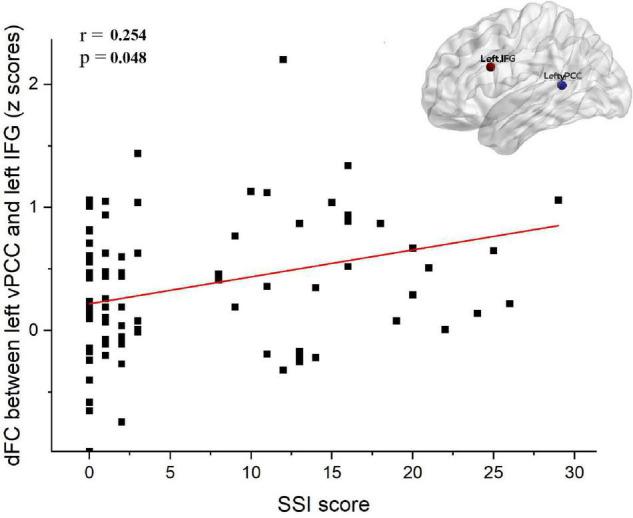
Correlation analysis between dFC and SSI score in depressed subjects (i.e., SI group and NSI group). The dFC between the between left vPCC and left IFG was positively associated with SSI score (*r* = 0.254, Bonferroni-corrected *p* = 0.048). SI, major depressive patients with suicidal ideation; NSI, major depressive patients without suicidal ideation; dFC, dynamic functional connectivity; vPCC, ventral posterior cingulate cortex; IFG, inferior frontal gyrus; SSI, scale for suicide ideation.

The dFC variability between the left vPCC and left IFG was positively correlated with the SSI scores of all patients with MDD (i.e., the SI group and NSI group combined; *r* = 0.254, Bonferroni-corrected *p* = 0.048; Figure 4). However, no correlation was observed between dFC and SSI scores within the SI group (*r* = 0.102, *p* = 0.572) or within the NSI group (*r* = 0.020, *p* = 0.886). There were no significant correlations between HAMD without suicide scores and dFC variability between the left vPCC and left IFG in patients with MDD. Furthermore, no significant correlations were found between other significantly different dFC variability subregions and the scores of SSI and HAMD without suicide in patients with MDD.

### Validation Analysis

The results of the 30-TR sliding-window length analysis validated the main results (50 TRs; see Supplementary Figure 1 and Supplementary Table 1). However, no significant differences were observed with the 70-TR sliding-window length.

## Discussion

To our knowledge, this study is the first to report aberrant dFC variability of PCC subregions in MDD patients with SI. Aberrant dFC variability between the left vPCC and left IFG was observed in MDD patients with SI in comparison with those with MDD patients without SI, while dFC variability abnormalities between the left dPCC and left fusiform gyrus, right vPCC, and left IFG were detected in MDD patients with SI relative to HCs. Furthermore, we confirmed the relationship between dFC abnormalities of the vPCC subregion and SI severity in patients with MDD. Overall, our findings reveal alterations in dFC variability between brain regions and demonstrate that SI is linked to aberrant dFC variability in patients with MDD. Our data advance the understanding of the potential neurobiological mechanisms of MDD with SI and point to options for clinical diagnostic biomarkers in the future.

### Aberrant Dynamic Functional Connectivity Variability in dPCC

The PCC has previously been subdivided into dorsal and ventral regions on the basis of post-mortem cytology measurements (Vogt et al., 2006). Each of the PCC subregions has distinct cytoarchitecture, patterns of structural connectivity, and resting-state functional connectivity (Vogt and Laureys, 2005; Margulies et al., 2009). It was suggested that the dPCC plays an important role in visual space and executive control of behavior (Vogt et al., 2006). In the current study, relative to HCs, both MDD patients with and without SI showed higher dFC variability between the left dPCC and left fusiform gyrus. However, no significant difference was observed between MDD patients with SI and MDD patients without SI. Our findings reflect the pathological effect of MDD on altered dFC patterns. The dPCC and fusiform gyrus are consistently reported to be involved in many aspects of cognition, such as word recognition, processing of color information (Weiner and Grill-Spector, 2010), and attentional focus (Leech and Sharp, 2014). Since these brain regions play a major role in cognition, the disrupted dFC variability between the left dPCC and left fusiform gyrus might contribute to negative self-perceptions and confer negative emotions (Schniering and Rapee, 2004) in depressed individuals. Our results highlight the idea that the dFC of key brain regions (such as the dPCC and left fusiform gyrus) in patients with MDD might show abnormalities and thus constitute a neurophysiological basis for the decreased ability to react flexibly to external or internal cognitive demands (Hamilton et al., 2011; Hutchison et al., 2013). Scholars have consistently proposed an analogous viewpoint. For example, Luo et al. (2021) detected decreased temporal variability of the dynamic index of bilateral PCC in patients with MDD in comparison with HCs, while other recent studies have reported dynamic alterations in brain activity in the fusiform gyrus in patients with MDD (Hou et al., 2018; Xue et al., 2020; Zhang et al., 2021). Therefore, it is plausible to consider that the observed anomalous dFC between the dPCC and fusiform gyrus is a neurobiological feature of patients with MDD. In conclusion, our findings could further enhance our understanding of how dFC properties support normal brain functions in patients with MDD.

### Aberrant Dynamic Functional Connectivity Variability in vPCC

In the current study, when compared with HCs, MDD patients with SI showed higher dFC variability between the left dPCC and the left IFG and between the right vPCC and left IFG. Moreover, relative to MDD patients without SI, MDD patients with SI showed higher dFC between the left vPCC and left IFG. Our data suggest that disrupted dFC between the vPCC and IFG may provide clues to the representation of neurocognition in MDD patients with SI. The vPCC is at an intermediate stage of information processing between visual recognition and emotion-related substrate (Johnson et al., 2002; Uddin et al., 2005). Interestingly, deficits in interference processing and learning/memory constitute an enduring defect in information processing in MDD patients with SI (Keilp et al., 2014). A previous study indicated that MDD patients with suicidal thoughts and behaviors showed structural and functional abnormalities in the PCC (Dombrovski et al., 2013; Peng et al., 2014).

Our findings could also be interpreted from a broader perspective. It is well known that the vPCC plays a key role in the default mode network (responsible for the processing of rumination) (Leech et al., 2011), while the IFG is the center hub of the frontoparietal network (responsible for handling behavioral inhibition) (Corbetta and Shulman, 2002). Thus, aberrant dFC between the vPCC and IFG in MDD patients with SI could constitute a high-risk circumstance in which the SI is converted to lethal action *via* impaired top-down behavior inhibition and impulsive decision-making (Schmaal et al., 2020). Hence, we conclude that the observed abnormal dFC variability in the MDD patients reveals impaired connectivity between the default mode network and frontoparietal network, which might relate to the potential neurobiological mechanisms of SI. In line with our findings, experimental evidence demonstrates altered dFC between the default mode network and frontoparietal network in patients with MDD (Demirtas et al., 2016; Yao et al., 2019). Furthermore, Liao et al. (2018) quantified dynamic connectomic variability using topological properties in patients with MDD with SI and found that the topological properties of dynamic connectomics could not only distinguish MDD patients with and without SI but could also predict the degree of SI. Congruent with previous findings, we suggest that the aberrant dFC might be regarded as a neurobiological feature for use in predictive and diagnostic models in patients with MDD with SI.

### Correlations Between Aberrant Dynamic Functional Connectivity Variability and Clinical Variables

We confirmed an association between dFC variability in the left vPCC subregion and SI severity in patients with MDD. However, we observed no correlation between dFC variability in the left vPCC subregion and the scores of HAMD without suicide in the patients with MDD. With regard to our finding that dFC variability is associated with SI severity but not with MDD severity (measured by the HAMD score without the SI part), we speculate that this may reflect the substantial impact of SI on brain dysfunction, rather than the pathological effects of the disease. Our findings support the idea that SI severity is related to anomalous dFC variability in patients with MDD. Schmaal et al. (2020) reviewed neuroimaging investigations across different mental illnesses for brain function, structural, and molecular alterations showing associations with suicidal thoughts and behaviors. They found that brain dysfunctions particularly converged in brain areas processing visual recognition and emotion regulation, such as the vPCC. Analogously, Auerbach et al. (2021) reported that altered vPCC volume was associated with SI and non-suicidal self-injury. Overall, we expect that the anomalous dFC variability in the left vPCC subregion underlies an emotional imbalance in individuals with SI. Collectively, the anomalous dFC variability in the left vPCC subregion may reflect SI severity, rather than illness *per se*.

There are several limitations to the current study. First, our study is a cross-sectional analysis, which restricts causal interpretations and longitudinal tracking of SI. Second, we compared the dFC variability differences between HCs and MDD patients with or without SI but did not include MDD patients with SA, who frequently show PCC dysfunction. A previous study reported that young depressed patients with SA exhibited lower PCC gray matter volume relative to HCs (Peng et al., 2014). In addition, decreased activity was found in the PCC during cognitive control in patients with mood disorders with SA (Minzenberg et al., 2015), and patients with MDD with SA exhibited an increased PCC response relative to HCs during the viewing of knives (Kim Y. J. et al., 2017). Furthermore, MDD patients with SA exhibited increased functional connectivity between the dPCC and left IFG when compared with MDD patients but without SA (Kim Y. J. et al., 2017). Thus, it would be meaningful to conduct a direct comparison of the dFC of PCC subregions between patients with SI and those with SA. Third, we acknowledge that our findings must be interpreted with caution because of the relatively small sample size. Finally, because of limitations resulting from the small sample size, we could not confirm the relationship between the dFC of the left vPCC subregion and SI severity in MDD patients with and without SI. The robustness of the left vPCC-left IFG contribution to SI needs further validation. Future studies with greater sample sizes that include longitudinal designs and across different mental illnesses are needed to corroborate our findings.

## Conclusion

Using dFC variability analyses of PCC subregions, we found that MDD patients with SI showed higher dFC between the left PCC and left IFG than those with MDD without SI. Moreover, the dFC variability positively correlated with SSI scores within all patients with MDD. The observed dFC abnormalities between vPCC and IFG might provide a neural-network explanation for SI and may provide new clues on the potential neurophysiological mechanisms of MDD with SI.

## Data Availability Statement

The original contributions presented in this study are included in the article/Supplementary Material, further inquiries can be directed to the corresponding authors.

## Ethics Statement

The studies involving human participants were reviewed and approved by the Ethics Committees of Affiliated Brain Hospital of Guangzhou Medical University. The patients/participants provided their written informed consent to participate in this study.

## Author Contributions

WL: investigation, formal analysis, writing – original draft, and visualization. CW and XL: validation, methodology, and investigation. FZ, LF, YY, and HL: investigation. KW, GHL, JC, and GXL: conceptualization. YZ: validation, project administration, methodology, and investigation. YN: conceptualization, supervision, and writing – review and editing. All authors contributed to the article and approved the submitted version.

## Conflict of Interest

The authors declare that the research was conducted in the absence of any commercial or financial relationships that could be construed as a potential conflict of interest.

## Publisher’s Note

All claims expressed in this article are solely those of the authors and do not necessarily represent those of their affiliated organizations, or those of the publisher, the editors and the reviewers. Any product that may be evaluated in this article, or claim that may be made by its manufacturer, is not guaranteed or endorsed by the publisher.

## Footnotes

1
http://rfmri.org/DPARSF

2
http://www.brainnetome.org/

3
http://rfmri.org/dpabi
