# Supplementary material for: Aberrant Dynamic Functional Connectivity of Posterior Cingulate Cortex Subregions in Major Depressive Disorder With Suicidal Ideation
Source: Front Neurosci. 2022 Jul 19;16:937145. doi: 10.3389/fnins.2022.937145 (PMC9344055; doi:10.3389/fnins.2022.937145)
Supplement: Supplementary file 1 [file Data_Sheet_1.docx]

**Table S1.** The areas of significantly different dFC among the SI, NSI, and HCs group (voxel *p* < 0.005, cluster *p* < 0.0125, GRF corrected)

| Subregion | Significant regions | MNI coordinates | | | Voxel size(mm^^3^) | F-value |
| --- | --- | --- | --- | --- | --- | --- |
|  |  | X | Y | Z |  |  |
| Left vPCC | Left inferior frontal gyrus | -45 | 9 | 24 | 567 | 11.616 |
| Right vPCC | Left inferior frontal gyrus | -42 | 9 | 21 | 459 | 11.774 |

Abbreviations: SI, major depressive patients with suicidal ideation; NSI, major depressive patients without suicidal ideation; HCs, heathy controls; vPCC, ventral posterior cingulate cortex.


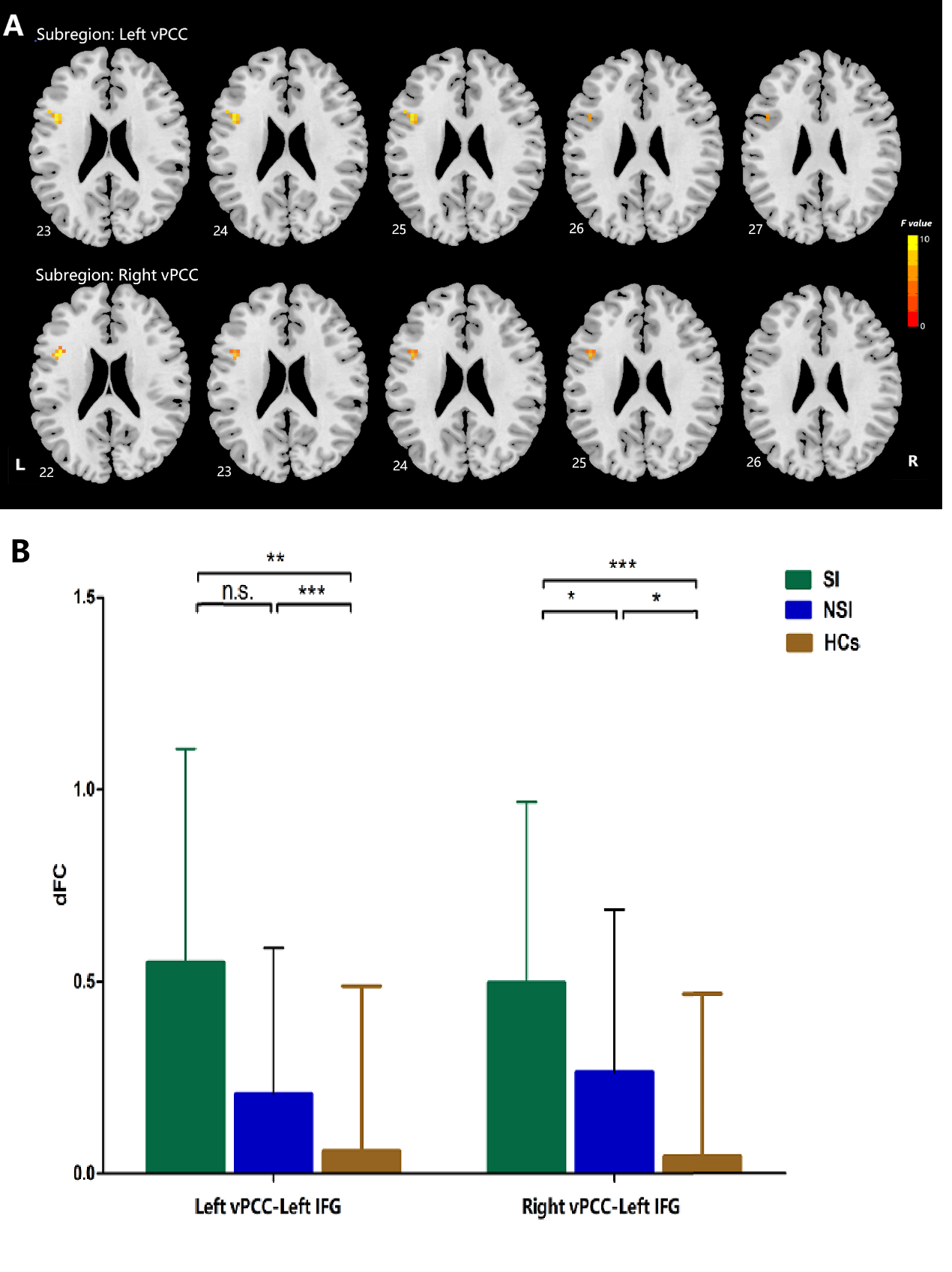


**Fig. S1.** ANCOVA analyses of dFC values among three groups when taking the subregion of posterior cingulate cortex as a seed. (A) Brain regions with significant differences among three groups, voxel *p* < 0.005, cluster *p* < 0.0125, GRF corrected. (B) Post hoc analyses of dFC values among three groups. Bonferroni corrected.

Abbreviations: dFC, dynamic functional connectivity; dPCC, dorsal posterior cingulate cortex; IFG, inferior frontal gyrus; SI, major depressive patients with suicidal ideation; NSI, major depressive patients without suicidal ideation; HCs, heathy controls. n.s., not significant.

* *p* < 0.05, ** *p* < 0.01, *** *p* < 0.001.
